# A New *Aspergillus fumigatus* Typing Method Based on Hypervariable Tandem Repeats Located within Exons of Surface Protein Coding Genes (TRESP)

**DOI:** 10.1371/journal.pone.0163869

**Published:** 2016-10-04

**Authors:** Rocio Garcia-Rubio, Horacio Gil, Maria Candida Monteiro, Teresa Pelaez, Emilia Mellado

**Affiliations:** 1 Mycology Deparment, Centro Nacional de Microbiologia, Instituto de Salud Carlos III (ISCIII), Majadahonda, Madrid, Spain; 2 Clinical Microbiology and Infectious Diseases Department, Hospital General Universitario Gregorio Marañon, Madrid, Spain; 3 European Program for Public Health Microbiology Training (EUPHEM), European Centre for Disease Prevention and Control, (ECDC), Stockholm, Sweden; Universidade de Sao Paulo, BRAZIL

## Abstract

*Aspergillus fumigatus* is a saprotrophic mold fungus ubiquitously found in the environment and is the most common species causing invasive aspergillosis in immunocompromised individuals. For *A*. *fumigatus* genotyping, the short tandem repeat method (STR*Af*) is widely accepted as the first choice. However, difficulties associated with PCR product size and required technology have encouraged the development of novel typing techniques. In this study, a new genotyping method based on hypervariable tandem repeats within exons of surface protein coding genes (TRESP) was designed. *A*. *fumigatus* isolates were characterized by PCR amplification and sequencing with a panel of three TRESP encoding genes: cell surface protein A; MP-2 antigenic galactomannan protein; and hypothetical protein with a CFEM domain. The allele sequence repeats of each of the three targets were combined to assign a specific genotype. For the evaluation of this method, 126 unrelated *A*. *fumigatus* strains were analyzed and 96 different genotypes were identified, showing a high level of discrimination [Simpson’s index of diversity (D) 0.994]. In addition, 49 azole resistant strains were analyzed identifying 26 genotypes and showing a lower D value (0.890) among them. This value could indicate that these resistant strains are closely related and share a common origin, although more studies are needed to confirm this hypothesis. In summary, a novel genotyping method for *A*. *fumigatus* has been developed which is reproducible, easy to perform, highly discriminatory and could be especially useful for studying outbreaks.

## Introduction

*Aspergillus* species are ubiquitous fungi which release huge amounts of spores into the air. Among them, *Aspergillus fumigatus* is the leading etiological agent of allergic and bronchopulmonary mycoses and is the most common species causing invasive aspergillosis in immunocompromised individuals [1]. Molecular typing methods have had an important impact in the aspergillosis field, including outbreak and reinfection investigations, patient and treatment monitoring, local and global epidemiology, and database construction [2]. The molecular analysis of the genetic and epidemiological relationship between environmental and clinical strains could allow for assessing potential strain origin and routes of transmission.

Over the years, many different molecular methods have been described for typing *A*. *fumigatus*, such as random amplified polymorphic DNA (RAPD) [3], amplified fragment length polymorphism analysis (AFLP) [4], restriction fragment length polymorphism analysis (RFLP) [5], and microsatellite length polymorphism (MLP) [6, 7]. However, these techniques show a poor inter-laboratory reproducibility. Retrotransposon insertion-site context (RISC) typing [8] and multilocus sequence typing (MLST) [9] present a good reproducibility and distinguish properly at genus and species level. Nevertheless, they have a low discriminatory power at subspecies level.

Currently, the reference typing method is the short tandem repeats of *A*. *fumigatus* assay (STR*Af*) based on microsatellite analysis [10]. Although this technique has superior discriminatory power, difficulties associated with sizing of the obtained PCR product and availability of the required laboratory technology encourage the development of novel, more accessible typing methods. Nowadays, due to the availability of sequencing and whole genome sequences, more simple approaches can be developed. Among them, a simple and rapid single-locus sequence typing method (CSP typing) is based on coding tandem repeats [11, 12] targeting the gene AfuA3g08990 which encodes a putative cell surface protein A (*csp*A). This method is a useful molecular tool although it has lower discriminatory power than microsatellite-based typing [11].

Some research has shown that several coding genes containing tandem repeats are exceptional genome dynamic components [12] which are very suitable targets for the development of new genotyping methods. The recombination events in these regions could cause changes in the number of repeats and alter the amino acid sequence of these proteins [13]. These changes are thought to be responsible for surface functional variability allowing quick adaptation to the host, evading its immune system and/or enhancing pathogenicity. Recently, some regions containing hypervariable tandem repeats located within exons of cell surface protein encoding genes have been identified in *A*. *fumigatus* [12]. These proteins are conserved in filamentous fungi and have no yeast homologs [14].

The purpose of this study was to establish a gene-typing panel using three targets based on three Tandem Repeats located within Exons of cell Surface Protein encoding genes (TRESP) with a high discriminatory power and able to differentiate epidemiologically unrelated *A*. *fumigatus* strains.

## Materials and Methods

### Aspergillus fumigatus strains

A total of 126 unrelated clinical strains of *A*. *fumigatus*, obtained from independent patients over a period of 19 years (1997–2015), were analyzed in this study. All of them belong to the strain collection of Spanish National Center of Microbiology or Hospital General Universitario Gregorio Marañon. All isolates were identified to the species level on the basis of PCR amplification and sequencing of ITS and β-tubulin genes [15]. In order to study the relationship between the discriminatory power obtained from the previously analyzed azole susceptible strains and those azole resistant, 49 azole resistant strains (27 with the TR_34_/L98H resistant mechanism and the remaining 22 showed other *cyp*51A related mechanisms) which belong to the described collections or were provided by international collaborations, were also characterized. Azole susceptible strains were mainly isolated from Spain; however, the origin of the resistant strains was variable: France, UK, the Netherlands, Denmark and Spain.

For typing purposes, *A*. *fumigatus* strains were subcultured in GYEP liquid medium (0.3% yeast extract, 1% peptone; BD-Difco, USA) with 2% glucose (Sigma-Aldrich, Spain) for 24 h at 37°C.

### TRESP typing panel

The three most variable TRESPs of the previously described *A*. *fumigatus* genes encoding repeat-rich cell wall or plasma membrane proteins [12] were selected in order to increase the discriminatory power of the method. These targets were: (i) Afu2g05150 encoding an MP-2 antigenic galactomannan protein (MP2), (ii) Afu6g14090 encoding hypothetical protein with a CFEM domain (CFEM), and (iii) Afu3g08990 encoding a cell surface protein A (CSP). This last target had been previously used for typing purposes [11]. The proposed typing nomenclature for MP2 and CFEM followed the described CSP’s structure [11]. The three targets are located in three different chromosomes [16] and are conserved among filamentous fungi, showing no significant homology to any yeast genes [14], which might be a result of the evolutionary distance between them [17].

### PCR amplification and sequencing of polymorphic loci

Genomic DNA was extracted using a procedure previously described [18]. Primers used to amplify a partial sequence of the three different genes are listed in S1 Table. Amplicon size was variable depending on the number of repeats in each strain. The PCR reaction mixtures contained 0.5 μM each primer, 0.2 μM deoxynucleoside triphosphate (Roche, Spain), 5 μl of PCR 10x buffer, 2 mM MgCl_2_, DMSO 5.2%, 2.5 U of Taq DNA polymerase (Applied Biosystems, California, USA), and 100–200 ng of DNA in a final volume of 50 μl.

The samples were amplified in a GeneAmp PCR System 9700 (Applied Biosystems) by using the following program parameters: 1 cycle of 5 min at 94°C and then 35 cycles of 30 s at 94°C, 45 s at 52 for MP2 gene or 54°C for CFEM gene, and 2 min at 72°C, followed by 1 final cycle of 5 min at 72°C. For amplifying CSP, the parameters were 1 cycle of 5 min at 94°C and then 35 cycles of 15 s at 94°C, 30 s at 62°C and 30 s at 68°C, followed by 1 final cycle of 5 min at 68°C.

The amplified products were purified using Illustra Exoprostar 1-step (GE Healthcare Life Science, UK) and both strands were sequenced with the Big-Dye terminator cycle sequencing kit (Applied Biosystems) following manufacturer instructions, using the same PCR amplification primers, with the exception of MP2 gene, in which a different set of primers (MP2.1 and MP2.2) was used (S1 Table).

### Sequence analysis: alleles and genotypes assignment

Sequences were assembled and edited using the Lasergene software package (DNAStar, Inc., Madison, WI). In order to identify the order and number of the tandem repeats in each gene, sequences were aligned using the multiple alignment fast Fournier transform method (MAFFT version 7) [19]. According to these repeats, alleles were assigned to each different sequence with the letter “m” for MP2 or “c” for CFEM followed by a correlative number. Alleles which varied in an individual nucleotide were differentiated adding a capital letter at the end (A, B). In the case of CSP, alleles were assigned according to the codes described previously [11, 20]. The new alleles described in this target were coded correlatively. The final genotype was obtained after combining the three alleles of each target, assigning a correlative genotype number for each new combination.

### Target reproducibility and stability

Two *A*. *fumigatus* reference strains (CM 237 and CBS 133.61) were chosen for studying the reproducibility and stability of the three selected targets. The long-term stability and reproducibility of the TRESP markers were estimated analyzing the typing results of both *A*. *fumigatus* strains that were alternatively subcultured and kept refrigerated (4°C) along 8 years (2007–2014), a minimum of 10 times over the years.

### Discriminatory power analysis of TRESP method

The discriminatory power of the method was estimated using Simpson’s index of diversity (D), as previously described [21]. This index was calculated for the unrelated azole susceptible strains. For inferring the effect of the three target combination over the discriminatory power of the method, this index was calculated separately for each target and also for the combination of the three.

### Genotypic diversity analysis

For each strain, a matrix with 140 positions was created according to the repeats or single nucleotide polymorphism (SNPs) present in each of the three TRESPs. In the matrix, the absence or presence of each repeat or SNP was indicated with a 0 or 1, respectively. In the cases that variation in the number of repeats was present, the number of these repeats was included in their specific positions. Afterwards, a clustering analysis in this matrix was performed using the Unweighted Pair Group Method Using Arithmetic Averages (UPGMA) and a dendrogram was built to infer the phylogenetic relationships of the strains, using InfoQuest™ FP 4.50 (BioRad, California, USA). Genotypic diversity was represented graphically using a minimum spanning tree (MST) generated using the combination of TRESP typing data.

## Results

### Reproducibility and stability of the targets

Overall the three targets showed an excellent reproducibility and stability. This was shown typing two reference strains that had been kept in the laboratory over eight years and which maintained the same sequence in each TRESP target in spite of the storage time or the number of times they were cultivated.

### Single allele sequence analysis

A total of 126 azole susceptible unrelated *A*. *fumigatus* isolates were analyzed combining the three TRESP targets (CSP, MP2 and CFEM) showing 100% typeability.

#### CSP typing: sequence analysis

We examined the amplified sequence from each strain considering the nucleotide sequence of each tandem repeat and the flanking regions (codons -15, -14, -1 and +1, +2, +3), as recommended in the proposed CSP type nomenclature [22]. All the 10 previously described repeat types [20, 22] were identified among the studied strains. The DNA sequence and amino acid translation of each repeat type are described in the S2 Table.

After analysis, a total of 21 CSP alleles were identified among all the strains, including 3 new alleles (t25, t26 and t27) not previously described (Table 1). Nine of the previously described CSP alleles were not found in this study (Table 1).

**Table 1:** Tandem repeats and flanking sequence for CSP types identified among 175 *A*. *fumigatus* isolates.

CSPAlleles	Codon			Tandem repeat succession	Codon			N°Strains (%)
	-15	-14	-1		+1	+2	+3	
t01	**GTG**	**GTC**	**CCG**	**01-01-01-01-----------------------05-03-01-06-03-07**	**CCA**	**CCT**	**CCA**	39 (22.3)
t09	**GTG**	**GTC**	**CCG**	**01-01-01-01-01-----------------------05-03-01-06-03-07**	**CCA**	**CCT**	**CCA**	2 (1.1)
t10	**GTG**	**GTC**	**CCG**	**01-01-01-----------------------05-03-01-06-03-07**	**CCA**	**CCT**	**CCA**	1 (0.6)
t18A	**GTG**	**GTC**	**CCG**	**01-01-----------------------05-03-01-06-03-07**	**CCA**	**CCT**	**CCA**	2 (1.1)
t18B	**G****C****G**	**C****TC**	**CCG**	**01-01-----------------------05-03-01-06-03-07**	**CCA**	**CCT**	**CCA**	1 (0.6)
t16^*^	**GTG**	**GTC**	**CCG**	**01-----------------------05-03-01-06-03-07**	**CCA**	**CCT**		0 (0.0)
t02	**GTG**	**GTC**	**CCG**	**01-01-02-03-04-----------------05-03-01-06-03-07**	**CCA**	**CCT**	**CCA**	32 (18.3)
t04A	**GTG**	**GTC**	**CCG**	**01-02-03-04-----------------05-03-01-06-03-07**	**CCA**	**CCT**	**CCA**	40 (22.9)
t04B	**GTG**	**GTC**	**CC****A**	**01-02-03-04-----------------05-03-01-06-03-07**	**CCA**	**CCT**	**CCA**	4 (2.3)
t06A	**GTG**	**GTC**	**CCG**	**01-01-01-02-03-04-----------------05-03-01-06-03-07**	**CC****A**	**CCT**	**CCA**	2 (1.1)
t06B	**GTG**	**C****TC**	**CCG**	**01-01-01-02-03-04-----------------05-03-01-06-03-07**	**CC****G**	**CCT**	**CC****T**	4 (2.3)
t14	**GTG**	**GTC**	**CCG**	**01-01-01-01-02-03-04-----------------05-03-01-06-03-07**	**CCA**	**CCT**	**CCA**	4 (2.3)
t12^*^	**GTG**	**GTC**	**CCG**	**01-01-01-01-01-02-03-04-----------------05-03-01-06-03-07**	**CCA**	**CCT**	**CCA**	0 (0.0)
t27^#^	**GTG**	**GTC**	**CCG**	**01-01-01-01-01-01-02-03-04-----------05-03-04-05-03-01-06-03-07**	**CC****G**	**CCT**	**CC****T**	1 (0.6)
t17^*^	**GTG**	**GTC**	**CCG**	**01-01-02-03-04-----------------05-03-09-06-03-07**	**CCA**	**CCT**	**CCA**	0 (0.0)
t03	**GTG**	**GTC**	**CCG**	**01-02-03-04-----------------------06-03-07**	**CCA**	**CCT**	**CCA**	19 (10.9)
t21^*^	**GTG**	**GTC**	**CCG**	**01-02-03-04-----------------------04-03-07**	**CCA**	**CCT**	**CCA**	0 (0.0)
t23^*^	**GTG**	**GTC**	**CCG**	**01-01-02-03-04-----------------------05-03-07**	**CCA**	**CCT**	**CCA**	0 (0.0)
t13	**GTG**	**C****TC**	**CCG**	**01-01-02-03-04-----------05-03-04-05-03-01-06-03-07**	**CC****G**	**CCT**	**CC****T**	2 (1.1)
t07^*^	**GTG**	**C****TC**	**CCG**	**01-02-03-04-----------05-03-04-05-03-01-06-03-07**	**CC****G**	**CCT**	**CC****T**	0 (0.0)
t08	**GTG**	**C****TC**	**CCG**	**01-01-01-02-03-04-----------05-03-04-05-03-01-06-03-07**	**CC****G**	**CCT**	**CC****T**	5 (2.9)
t15	**GTG**	**C****TC**	**CCG**	**01-01-01-01-02-03-04-----------05-03-04-05-03-01-06-03-07**	**CC****G**	**CCT**	**CC****T**	1 (0.6)
t19	**GTG**	**C****TC**	**CCG**	**01-01-02-03---10-----03-04-05-03-04-05-03-01-06-03-07**	**CC****G**	**CCT**	**CC****T**	2 (1.1)
t25^#^	**GTG**	**GTC**	**CCG**	**01---------------------06-03-07**	**CCA**	**CCT**	**CCA**	1 (0.6)
t24^*^	**GTG**	**GTC**	**CCG**	**01-01-01-01-03-01---------------------06-03-07**	**CCA**	**CCT**	**CCA**	0 (0.0)
t05	**GTG**	**GTC**	**CCG**	**01-01-01-03-01---------------------06-03-07**	**CCA**	**CCT**	**CCA**	5 (2.9)
t11	**GCG**	**C****TC**	**CCG**	**01-01-08-03-01---------------------06-03-07**	**CCA**	**CCT**	**CCA**	7 (4.0)
t22^*^	**GTG**	**GTC**	**CCG**	**01-01-02-03-01---------------------06-03-07**	**CCA**	**CCT**	**CCA**	0 (0.0)
t26^#^	**GTG**	**GTC**	**CCG**	**01-01-------------------------------03-07**	**CCA**	**CCT**	**CCA**	1 (0.6)
t20^*^	**GTG**	**GTC**	**CCG**	**01-02-------------------------------03-07**	**CCA**	**CCT**		0 (0.0)

CSP alleles not found (*) and first described (^#^) in this study. Number of strains and percentage of each allele are indicated on the right.

The frequency of each CSP allele was variable (Table 1), t04A (22.9%) and t01 (22.3%) being predominant alleles within all strains. However, some CSP alleles were considerably less represented, such as t10, t15, t18B, along with the newly described t25, t26 and t27, which were identified in single strains. The discriminatory power (D) of this marker using our strain collection of unrelated *A*. *fumigatus* resulted in 0.854 (Table 2).

**Table 2:** Summary data for TRESP typing method using unrelated strains.

Genes	Number of genotypes	Simpson’s index of diversity (D)
CSP	20	0.854
MP2	32	0.852
CFEM	22	0.851
**TRESP**	**96**	**0.994**

Simpson’s index of diversity (D) of each target alone and in combination (TRESP) of 126 azole susceptible strains.

#### MP2 typing: sequence analysis

The sequence of each strain was analyzed considering the nucleotide sequence of each tandem repeat in this gene. The MP2 tandem repeat sequences are formed mainly by 39-mer repeats [12]. Each tandem repeat sequence could be divided into two parts, the first 18 bp invariably starts with a particular sequence (GAGA or GAAA) and the second 21 bp begins always with GAGA (S3 Table). In addition, a short repeat (sr) of 18 bp, which only follows the first part of the structure described above, was found in some isolates. In total, 27 different MP2 repeats were found (S3 Table). After some specific repeats (r02, r08, r11, r17, and r22) short sequences of 6 or 9 nucleotides appeared encoding several prolines (PP, PPP) and sometimes followed by a threonine (PPT) (Table 3).

**Table 3:** MP2 alleles identified among 175 *A*. *fumigatus* isolates.

MP2 Alleles	Tandem repeat succession	N° strains (%)
m1.1	**01-02-[P-P-P]-01-03-04-05-06--------------------07-08-[P-P-P]-09---------04-10-11-[P-P]**	73 (41.7)
m1.2	**01-02-[P-P-P]-01---04-05-06--------------------07-08-[P-P-P]-09---------04-10-11-[P-P]**	9 (5.1)
m1.3	**01-02-[P-P-P]-01-----05-06--------------------07-08-[P-P-P]-09---------04-10-11-[P-P]**	4 (2.3)
m1.4	**01-02-[P-P-P]-------05-06--------------------07-08-[P-P-P]-09---------04-10-11-[P-P]**	1 (0.6)
m1.5	**01-02-[P-P-P]-01---04---06--------------------07-08-[P-P-P]-09---------04-10-11-[P-P]**	3 (1.7)
m1.6	**01-02-[P-P-P]-01-----05-06--------------------07-08-[P-P-P]-09-----------10-11-[P-P]**	1 (0.6)
m1.7	**--02-[P-P-P]-01-03-04-05-06--------------------07-08-[P-P-P]-09---------04-10-11-[P-P]**	1 (0.6)
m1.8	**----------01-03-04-05-06--------------------07-08-[P-P-P]-09---------04-10-11-[P-P]**	2 (1.1)
m1.9	**01-02-[P-P-P]-01-03-04-05-06--------------------07-08-[P-P-P]-------01-03-04-10-11-[P-P]**	2 (1.1)
m2.1	**01-02-[P-P-P]-01-03-04---06-12-13-4-6-12-02-[P-P-P]-14--07-08-[P-P-P]-09---------04-10-11-[P-P]**	1 (0.6)
m2.2	**01-02-[P-P-P]-01-03-04---06-12--------02-[P-P-P]-14--07-08-[P-P-P]-09---------04-10-11-[P-P]**	2 (1.1)
m3.1	**03----------------------------------------------------------03-22-[P-P]**	1 (0.6)
m3.2	**03--------------------------------15-20-15-S-21---15--------------03-22-[P-P]**	1 (0.6)
m3.3	**03----------------05-03-------------15-20-15-S-21---15--------------03-22-[P-P]**	1 (0.6)
m3.4	**03-15--------------05-03-------------15-20-15-S-21---15--------------03-22-[P-P]**	14 (8.0)
m3.5	**03-15-20-15-S-21-15-03-15-05-03-------------15-20-15-S-21---15--------------03-22-[P-P]**	2 (1.1)
m3.6	**03-15--------------05-03-------------15-20-15-S-21-06-15-20-15-S-21-15-S-21-03-22-[P-P]**	1 (0.6)
m3.7	**03-15-20--------------------------26-15-20-15-S-21---15--------------03-22-[P-P]**	1 (0.6)
m4.1	**--------------03---05-03-------------15-20-15-S-21---15-20-15-S-21-15----03-17-[P-P]**	1 (0.6)
m4.2	**--------------03---05-03-------------15-20-15-S-21------------15----03-17-[P-P]**	1 (0.6)
m5.1	**03-15-05-03-15------------------------S-S-S-16-15-03------------15------17-[P-P]**	4 (2.3)
m5.2	**03-15-05-03-15--------------------------S-S-16-15-03------------15------17-[P-P]**	1 (0.6)
m5.3	**03-15-05-03-15---------------------------S-16-15-03------------15------17-[P-P]**	21 (12.0)
m5.4	**03-15-05-03-15----------------------------16-15-03------------15------17-[P-P]**	7 (4.0)
m5.5	**03-15-05-03-15------------------------16-15-16-15-03------------15------17-[P-P]**	5 (2.9)
m5.6	**03-15-05-03-15----------------------------------------------------17-[P-P]**	1 (0.6)
m5.7	**03--------------------------------25-----15-03------------15------17-[P-P]**	1 (0.6)
m6.1	**03-15-05-03-15-05-03-15-05-03-15-05-03-15-05----16--------------------15------17-[P-P]**	1 (0.6)
m6.2	**03-15-05-03-15-05-03-15-05-03-15------------16-24-16-15---------------------17-[P-P]**	1 (0.6)
m6.3	**03-15------------------------------16-15-16-15---------------------17-[P-P]**	1 (0.6)
m7.1	**18------08-[P-P-P]-19----------02-[P-P-P]-01-03-07-08-[P-P-P]-07------------23-11-[P-P]**	5 (2.9)
m8.1	**--------------------------03-02-[P-P-P]-01-03-07-08-[P-P-T]-07-02-[P-P-P]-19-23-11-[P-P]**	1 (0.6)
m9.1	**-------------------------------------03-07-08-[P-P-T]-07----------23-11-[P-P]**	3 (1.7)
m10.1	**07------08-[P-P-P]----------------------01-03-07-08-[P-P-T]-07----------23-11-[P-P]**	1 (0.6)

Number of strains of each allele and percentage are indicated on the right.

Analyzing the number and order of the repeats, 34 MP2 alleles were identified all strains (Table 3). The highest frequency was presented in m1.1 allele (41.7%), followed by m3.4 (8%) and m5.3 (12%). Nineteen of the MP2 alleles were identified in single strains (Table 3). The D of the typing technique using only MP2 gene resulted in 0.852 among the 126 unrelated isolates (Table 2).

#### CFEM typing: sequence analysis

Similar to CSP and MP2, CFEM was analyzed considering each tandem repeat nucleotide sequence. Each tandem repeat sequence is formed by a CFEM 6-mer repeats [12] coding a particular sequence of amino acids, which starts with a serine in most of the cases (S4 Table). We found a different type of repeat (r11), which did not follow the described structure, formed by 12 bp and encoding the amino acids TATG (S4 Table). In total, 13 different repeats were found (S4 Table). Moreover, there is a region of 21 bp without repeats between r11 and r12 in all strains. To increase discrimination ability, four SNPs were also considered: SNP122, SNP155, SNP685 and SNP815.

Analyzing this structure, a total of 23 CFEM alleles were identified (Table 4). The CFEM allele (c08A) was found in 52 strains (29.7%), followed by c09 which was found in 42 strains (24%) (Table 4). The D of the typing technique using CFEM gene was 0.851 for unrelated strains (Table 2).

**Table 4:** CFEM alleles identified among 175 *A*. *fumigatus* isolates.

CFEM Alelles	Tandem repeat succession	N° R11	N° TRs (bp)	N° R12	N° Strains (%)
c01	06-01-01-01---02-02-01-03-03-04-03-05-------04-03-03-03-06-01-04-03-05-07-03-04-03-08-01-03-09-10	6	21	4	3 (1.7)
c02	06-01-01-01---02-01-03-03-04-03-05---------04-03-03-03-06-01-04-03-05-07-03-04-03-08-01-03-09-10	7	21	4	3 (1.7)
c03	06-01-01-01---02-01-03-03-04-03-05---------04-03-03-03-06-01-04-03-05-07-03-04-03-08-01-03-09-10	5	21	4	4 (2.3)
c04	06-01-01-01---02-01-03-03-04-03-05---------04-03-03-03-06-01-04-03-05-07-03-04-03-08-01-03-09-10	4	21	4	9 (5.1)
c05A	06-01-01-01---02-01-03-03-04-03-05---------04-03-03-03-06-01-04-03-05-07-03-04-03-08-01-03-09-10	3	21	5	16 (9.1)
c05B^#^	06-01-01-01---02-01-03-03-04-03-05---------04-03-03-03-06-01-04-03-05-07-03-04-03-08-01-03-09-10	3	21	5	2 (1.1)
c06	06-01-01-01---02-01-03-03-04-03-05---------04-03-03-03-06-01-04-03-----------08-01-03-09-10	3	21	5	2 (1.1)
c07	06-01-01-01---02-01-------03-05---------04-03-03-03-06-01-04-03-05-07-03-04-03-08-01-03-09-10	3	21	4	4 (2.3)
c08A	06-01-01-01---02-01-03-03-04-03-05---------04-03-03-03-06-01-04-03-05-07-03-04-03-08-01-03-09-10	3	21	4	52 (29.7)
c08B^$^	06-01-01-01---02-01-03-03-04-03-05---------04-03-03-03-06-01-04-03-05-07-03-04-03-08-01-03-09-10	3	21	4	5 (2.9)
c09	06-01-01-01---02-01-03-03-04-03-05---------04-03-03-03-06-01-04-03-05-07-03-04-03-08-01-03-09-10	2	21	4	42 (24.0)
c10^&^	06-01-01-01---02-01-03-03-04-03-05---------04-03-03-03-06-----------------------09-10	2	21	4	7 (4.0)
c11*	06-01-01-01---02-01-03-03-04-03-05---------------03-06-------------------01-03-09-10	4	21	4	6 (3.4)
c12	06-01-01-01---02-01---------------------03-03-03-06-01-04-03-05-07-03-04-03-08-01-03-09-10	3	21	4	5 (2.9)
c13*	06-01-01-01---02-01-03-03-04-03-05---------------------------------------03-09-10	2	21	4	1 (0.6)
c14*	06-01-01-01---02-01-03-03-04-03-05---------------------------------------03-09-10	3	21	4	1 (0.6)
c15	06-01-01-01---02-01-03-03-04-03-05---------------03-06-------------------01-03-09-10	6	21	4	1 (0.6)
c16	06-01-01-01---02-01-03-03-04-03-05---------04-03-03-03-06-13-04-03-05-07-03-04-03-08-01-03-09-10	2	21	4	4 (2.3)
c17	06-01-01-01---02-01-03-03-04-03-05---------04-03-03-03-06-01-04-03-05-07-03-04-03-08-01-03-09-10	2	21	5	2 (1.1)
c18	06-01-01-01---02-01-03-03-04-03-05---------04-03-03-03-06-01-04-04-05-07-03-04-03-08-01-03-09-10	2	21	4	1 (0.6)
c19	06-01-01-01-01-02-01-03-03-04-03-05-04-03-03-03-04-03-03-03-06-------------------01-03-09-10	4	21	3	3 (1.7)
c20	06-01-01-01---02-01-03-03-04-03---------------03-03-06-01-04-03-05-07-03-04-03-08-01-03-09-10	3	21	4	1 (0.6)
c21	06-01-01-01---02-01-03-03-04-03-05---------------03-06-------------------01-03-09-10	3	21	4	1 (0.6)

Number of strains and percentage of each allele are indicated on the right.

Single nucleotide polymorphism (SNP)

^#^SNP122 g/a

^*^SNP155 a/g

^&^SNP685 t/a and

^$^SNP815 a/t. SNP positions were determined according to the whole genome sequence of *Aspergillus fumigatus Af*293 (GenBank accession number CM000174.1), where CFEM domain protein starts in 3593051 to 3594095 bp.

### TRESP genotyping analysis of unrelated strains

Combining the results obtained from the three alleles, a total of 96 genotypes were identified among the 126 *A*. *fumigatus* unrelated strains included in the study (S5 Table). Among them, 77 genotypes were represented by a single isolate, and considering the remaining the most commonly observed genotype was t02m1.1c09 (n = 5 isolates) (S5 Table). The discriminatory power calculated for the three genes individually were 0.854 when only using CSP, 0.852 using MP2 and 0.851 using CFEM. D value calculated for the combination of the three alleles was 0.994 (Table 2).

### TRESP genotyping analysis: azole susceptible vs azole resistant strains

In contrast with the 20 CSP alleles found among the azole susceptible strains, only 6 CSP alleles (t01, t02, t03, t04A, t04B, and t11) were found among the azole resistant strains (n = 49). D value of this marker was 0.754 for this group of strains.

Similarly, using MP2 gene, 32 alleles were found among azole susceptible isolates. However, only 7 MP2 alleles (m1.1, m1.2, m1.4, m1.5, m3.4, m5.1 and m5.3) were obtained in the azole resistant group. Interestingly, among TR_34_/L98H resistant strains only 4 alleles were found (m1.1, m1.2, m1.4 and m1.5), and two of them were unique for this group (m1.4 and m1.5). MP2 D value was 0.616 for resistant strains.

Twenty two CFEM alleles were found among all azole susceptible strains and nine alleles for azole resistant strains. TR_34_/L98H isolates were distributed in five CFEM alleles (c04, c08A, c08B, c09 and c12). However, these CFEM types included azole susceptible and TR_34_/L98H resistant isolates. Resistant strains, excluding TR_34_/L98H isolates, belonged to eight CFEM alleles (c04, c05A, c06, c07, c08A, c08B, c09 and g18). The D of the typing technique using CFEM gene was 0.711 for the resistant strains.

D value calculated for the combination of the three genes was 0.994 among unrelated strains and they were represented by 96 genotypes. Among them, very few genotypes were represented by more than one strain. However, resistant isolates were represented by only 26 genotypes and had a lower TRESP D (0.890). The number of genotypes decreased to 12 when TR_34_/L98H resistant strains were analyzed, with the genotype t02m1.1c09 identified in 13 of the 27 isolates.

**Fig 1:**
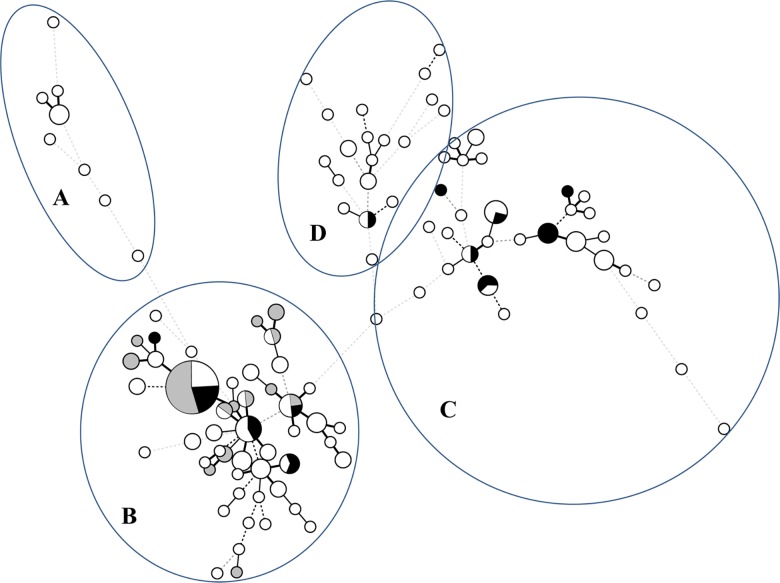
Minimum spanning tree (MST) showing the genotypic relationship between the azole-resistant and azole-susceptible *A*. *fumigatus* isolates. Each circle corresponds to a unique genotype, and the size of the circle proportionally represents the number of isolates with that genotype (1 to 21). Connecting lines correspond to the number of differences between the genotypes. Short bold line, 1 difference; black line, 2 differences; long grey line, 3 differences; dotted line, 4 or more differences. Grey circles, azole resistant TR_34_/L98H, (n = 27); black circles, azole resistant non TR_34_/L98H, (n = 22); white circles, azole susceptible strains, (n = 126). Four clusters were found (A-D).

Genotypic diversity was graphically represented using a minimum spanning tree (MST) based on the combination of the three TRESP alleles (Fig 1). Four big clusters (Fig 1, clusters A-D) could be identified in the MST. Notably, all TR_34_/L98H resistant strains (independently of their geographical origin) were represented in a cluster (B) which also included some azole susceptible and resistant strains non-TR_34_/L98H. On the other hand, resistant isolates with other *cyp*51A related resistance mechanisms were distributed in three clusters along with susceptible strains (cluster B, C and D). Cluster A was exclusively form by susceptible isolates. The dendrogram derived from the TRESP typing analysis which contains all the strains included in this study is shown in S1 Fig. Similar to the MST, strains were distributed in four different clades (A-D), with the TR_34_/L98H resistant strains located exclusively in the clade B.

## Discussion

Over the years, many different molecular methods have been described for typing *A*. *fumigatus* [9, 11, 23, 24, 25]. Among them, the reliable gold standard method (STR*Af*) based on microsatellite analysis proved to be an extremely robust typing assay with a very high discriminatory power [10]. However, various methodological difficulties have encouraged the development of novel typing techniques, although up to date they have shown a lower discriminatory power compare with STR*Af*.

The aim of this study was to establish a novel genotyping method able to differentiate epidemiologically unrelated *A*. *fumigatus* strains. TRESP typing is based on PCR amplification and sequencing of three targets, with tandem repeat variations in protein-coding regions, which present a large diversity in repeat-encoded structures [12].

TRESP typing presented a good reproducibility and stability as has been shown with the two reference strains, indicating that alleles are stable to allow epidemiological studies. In addition, TRESP has allowed us to identify 96 genotypes among the 126 unrelated azole susceptible strains, showing a good discrimination power (D = 0.994). Since MP2 and CFEM have been suggested for typing purposes in this work, comparison with other studies is lacking except for CSP results, which could be compared with previous studies. Azole susceptible strains were distributed in 20 different CSP groups, similar to previously published [26]. Comparing our CSP D value with other studies, it was similar to the range described previously for this method (0.800–0.832) [20, 22, 27]. The most common CSP alleles and lineages in this study are also the most usual ones in other studies of different countries such as USA, The Netherlands, Australia and China [11, 20, 22, 27]. This supports the fact that *A*. *fumigatus* has a cosmopolitan distribution and a lack of geographically distinctive *A*. *fumigatus* populations, recently described by using Whole-Genome Sequencing analysis [28]. When CSP was analyzed using only azole resistant strains with TR_34_/L98H, five CSP alleles were found, including two types never described among TR_34_/L98H strains (t01, t04A) [26]. In our work, there was only one CSP allele (t04B) in which only resistant strains with TR_34_/L98H were found. Moreover, t11 CSP allele was found in both susceptible and resistant strains, contrary to previously described [26].

The other two targets (MP2 and CFEM) results resemble those obtained with CSP. Comparing D value of the available typing techniques, TRESP method is a competitive strategy to discriminate among *A*. *fumigatus* strains. In published guidelines for typing methods, a D≥0.95 is recommended [29]. The STR*Af* discrimination power, highlighting that it is the gold standard method, was 0.9994 when nine markers were used and 0.9968 for a single locus (STR*Af* 3A) [10]. However, in other works, D value yielded with this method was variable: (0.9968 [10], 0.988–0.995 [30], 0.984 [31], and 0.9994 [32]) probably depending on the characteristics of the study and the variability of the analysis conditions. Recently, there has been a growing interest in developing more accessible typing tools due to the technical difficulties of the gold standard method, searching for new typing approaches which will require only the ability to perform PCR and having access to an automated sequencer. An example of this is MLST assay which performs well at the genus and species level with *Candida*, have a D value far from good (D = 0.93) in the case of *Aspergillus* [9].

From a methodological point of view, the most important key element in the use of microsatellites, and particularly in STR*Af*, is to translate the fragment electrophoretic mobility to their repeat number using high-resolution equipment such as capillary-based or acrylamide-based electrophoresis platforms. However, this mobility is dependent on many critical factors such as the presence, or not, of denaturing compounds, the matrix, the run temperature, the sequence of the fragment, the florescent labels, the sizing marker, etc [2]. In order to get exchangeable typing results between laboratories, it is necessary to run allelic ladders for calibrating every platform [33]. Also, specialized and expensive equipment, dedicated software and personnel specifically trained for that assay are needed for this technique. In contrast, TRESP typing can be successfully used for *A*. *fumigatus* typing as it only requires the ability to perform PCR and access to an automated sequencer. The high reproducibility and stability of the TRESP assay and the easy exchange of the results will allow an optimal interlaboratory comparison of data. This approach, exclusively based on sequencing data, has the advantage of accurate database development and is totally reliable for taxonomy. Also, its quickness and simplicity, not requiring elaborate training or special software for analysis, facilitates its integration into any clinical microbiology laboratory.

In addition, the TRESP method was used to compare the genetic diversity between azole susceptible and resistant strains allowing for speculation about the origin and spread of strains with TR_34_/L98H azole resistance genotype within Europe. Analyzing TRESP typing results, there is strong evidence that susceptible strains have a greater genetic diversity compared to resistant strains, particularly to those strains with TR_34_/L98H (Fig 1 and S1 Fig). This idea is supported by TRESP typing results, first, due to the appearance of the TR_34_/L98H resistant strains in a unique cluster in both MST and dendrogram, in contrast to the wide distribution of the non-TR_34_/L98H resistant isolates which were dispersed in three different clusters; and second, the different amount of total genotypes between susceptible and resistant strains (S5 Table). A total of 96 TRESP genotypes were found for susceptible strains, while TR_34_/L98H resistant isolates are grouped in only 12 genotypes, despite their multiple geographic origin. This poor TRESP genotype diversity of TR_34_/L98H isolates indicates how unlikely it is that TR_34_/L98H mechanism emerged recurrently and independently, as it has been previously suggested [26]. A possible explanation would be the selection of TR_34_/L98H resistance mechanism from a common ancestor or reduced set of related isolates with short genetic distances as has been previously suggested [26, 34]. Alternatively, differences in resistance mechanism acquisitions could be related to some specific genotypes, a new research area that we are further exploring.

In summary, this study demonstrates that TRESP typing is a novel genotyping method for *A*. *fumigatus* that fulfils all the needs of a suitable typing strategy with a high competitive discriminatory power and that could be especially useful for studying outbreaks.

## Supporting Information

S1 Fig Dendrogram based on TRESP results from 175 *A*. *fumigatus* strains. Name of each strain, origin, azole resistance mechanism and TRESP identification number (TRESP ID) are shown. The scale bar indicates the percentage identity. Four clusters were found (A-D).
(TIF)

S1 Table Oligonucleotides used for amplification and sequencing the three target genes: CSP, MP2 and CFEM. (DOCX)

S2 Table CSP repeat types. Nucleotide and amino acid sequences among 175 *A*. *fumigatus* strains.
(DOCX)

S3 Table MP2 repeat types: nucleotide and amino acid sequences identified among 175 *A*. *fumigatus* isolates. (DOCX)

S4 Table CFEM repeat types: nucleotide and amino acid sequences identified among 175 *A*. *fumigatus* isolates. (DOCX)

S5 Table Total TRESP genotypes. TRESP ID, number of strains of each type and proportion.
(DOCX)

S6 Table GenBank Accesion Numbers of all TRESP types. (DOCX)
