# Supplementary figures and images for: A New Aspergillus fumigatus Typing Method Based on Hypervariable Tandem Repeats Located within Exons of Surface Protein Coding Genes (TRESP)
Source: PLoS One. 2016 Oct 4;11(10):e0163869. doi: 10.1371/journal.pone.0163869 (PMC5049851; doi:10.1371/journal.pone.0163869)

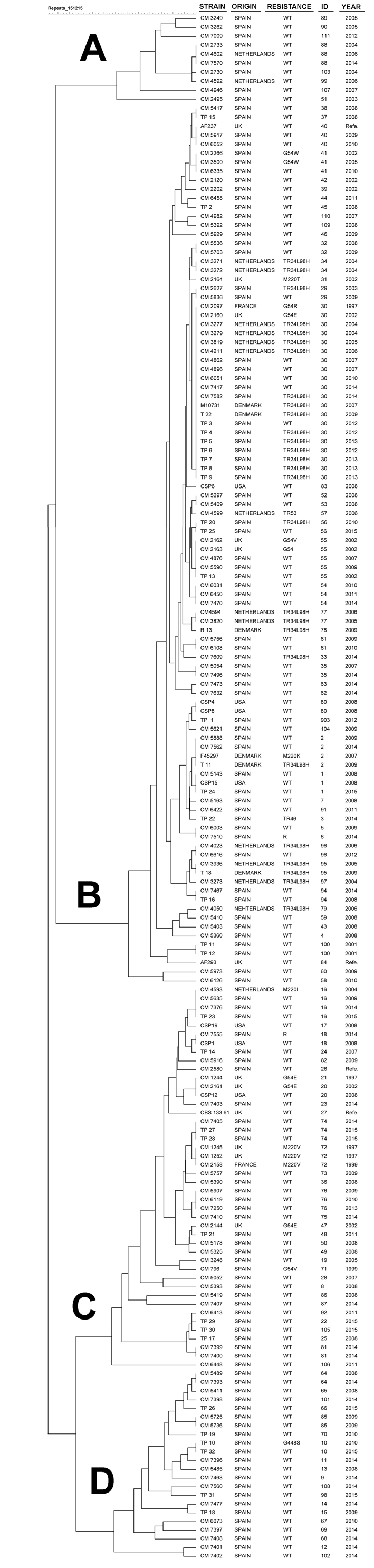

Supplement: S1 Fig — Name of each strain, origin, azole resistance mechanism and TRESP identification number (TRESP ID) are shown. The scale bar indicates the percentage identity. Four clusters were found (A-D). (TIF) [file pone.0163869.s001.tif]
