# Supplementary material for: A New Aspergillus fumigatus Typing Method Based on Hypervariable Tandem Repeats Located within Exons of Surface Protein Coding Genes (TRESP)
Source: PLoS One. 2016 Oct 4;11(10):e0163869. doi: 10.1371/journal.pone.0163869 (PMC5049851; doi:10.1371/journal.pone.0163869)
Supplement: S1 Table — (DOCX) [file pone.0163869.s002.docx]

**S1 Table.** **Oligonucleotides used for amplification and sequencing the three target genes: CSP, MP2 and CFEM.**

| Genes | Primers (5’-3’) | | Amplicon size (bp) | Origin |
| --- | --- | --- | --- | --- |
| CSP | CSP1F | TTGGGTGGCATTGTGCCAA | 1156-1276 | [11] |
|  | CSP2R | GAGCATGACAACCCAGATACCA |  |  |
| MP2 | MP2A | ATGCGGTTCTCTGCGTTA | 1128-1779 | This study |
|  | MP2B | CAGCAACAGTGCAAATGC |  |  |
|  | MP2.1* | CTCGAACTTGGCTACGAC | 404-1055 | This study |
|  | MP2.2* | AGGTAGTGGAGGTCACTG |  |  |
| CFEM | CFEMA | ATGAAGGCCTCTGTGTC | 918-1044 | This study |
|  | CFEMB | AGGATAATCAAGGCAGCG |  |  |

*Used only for MP2 sequencing
