# Supplementary material for: A New Aspergillus fumigatus Typing Method Based on Hypervariable Tandem Repeats Located within Exons of Surface Protein Coding Genes (TRESP)
Source: PLoS One. 2016 Oct 4;11(10):e0163869. doi: 10.1371/journal.pone.0163869 (PMC5049851; doi:10.1371/journal.pone.0163869)
Supplement: S2 Table — Nucleotide and amino acid sequences among 175 A. fumigatus strains. (DOCX) [file pone.0163869.s003.docx]

**S2 Table. CSP repeat types: nucleotide and amino acid sequences identified among 175 *A. fumigatus* isolates.**

| CSP Repeat type | Repeat sequence | Amino acid sequence |
| --- | --- | --- |
| r01 | ACT TCT GTC CCG | T S V P |
| r02 | ACT TCT GTC CCA | T S V P |
| r03 | ACT CAA AAC GCG | T Q N A |
| r04 | ACT TCA ATC CCG | T S I P |
| r05 | ACT TTT GTC CCG | T F V P |
| r06 | ACT TCA GTC CCG | T S V P |
| r07 | ACT ACT ATT GTG | T T I V |
| r08 | ACT TTT CTC CCG | T F L P |
| r09 | ACT TCT GTT CCG | T S V P |
| r10 | ACT TCA ATC CCA | T S I P |
