# Supplementary material for: A New Aspergillus fumigatus Typing Method Based on Hypervariable Tandem Repeats Located within Exons of Surface Protein Coding Genes (TRESP)
Source: PLoS One. 2016 Oct 4;11(10):e0163869. doi: 10.1371/journal.pone.0163869 (PMC5049851; doi:10.1371/journal.pone.0163869)
Supplement: S3 Table — (DOCX) [file pone.0163869.s004.docx]

**S3 Table. MP2 repeat types: nucleotide and amino acid sequences identified among 175 *A. fumigatus* isolates.**

| MP2 Repeat type | Repeat sequence | Amino acid sequence |
| --- | --- | --- |
| r01 | **GAGA**CCTCCACTCCGTGC**GAGA**CTACTACCACTACTACC | ETSTPCETTTTTT |
| r02 | **GAGA**CCTCTACCCCCTGT**GAGA**CTACTACTACGACTACT |  |
| r08 | **GAAA**CCTCTACCCCCTGT**GAGA**CTACTACTACGACTACT |  |
| r11 | **GAAA**CCTCCACTCCATGT**GAGA**CTACCACCACTACTACA |  |
| r12 | **GAGA**CCTCTACCCCCTGT**GAGA**CTACTACCACTACTACC |  |
| r17 | **GAGA**CCTCTACTCCGTGT**GAGA**CTACCACCACTACTACA |  |
| r22 | **GAGA**CCTCTACTCCGTGT**GAGA**CTACTACCACTACTACA |  |
| r03 | **GAGA**CCTCCACTCCATGT**GAGA**CCACTACCACTCCTACC | ETSTPCETTTTPT |
| r04 | **GAGA**CCTCTACCCCCTGT**GAGA**CTACTACTACCCCTACC |  |
| r05 | **GAGA**CCTCTACTCCGTGT**GAGA**CTACCACCACTCCTACC |  |
| r06 | **GAGA**CCTCTACTCCGTGT**GAGA**CCACTACCACTCCTACC |  |
| r07 | **GAGA**CCTCTACTCCATGC**GAGA**CTACCACCACTCCTACC |  |
| r09 | **GAGA**CCTCCACTCCGTGC**GAGA**CCACTACCACTCCTACC |  |
| r13 | **GAGA**CCTCTACCCCCTGT**GAGA**CTACTACCACTCCTACC |  |
| r14 | **GAGA**CCTCCACTCCGTGT**GAGA**CCACTACCACTCCTACC |  |
| r15 | **GAGA**CCTCTACTCCATGC**GAGA**CTACTACTACTCCTACC |  |
| r18 | **GAGA**CCTCCACTCCATGC**GAGA**CTACCACCACTCCTACC |  |
| r19 | **GAGA**CCTCCACTCCGTGC**GAGA**CTACTACCACTCCTACC |  |
| r25 | **GAGA**CCTCTACTCCATGC**GAGA**CTACTACCACTCCTACC |  |
| r26 | **GAGA**CCTCTACTCCATGT**GAGA**CCACTACCACTCCTACC |  |
| r10 | **GAGA**CCTCTACTCCGTGT**GAGA**CTACTACTACTCCTCCT | ETSTPCETTTTPP |
| r23 | **GAGA**CCTCTACCCCCTGT**GAGA**CTACTACTACTCCTCCT |  |
| r16 | **GAGA**CCTCTACTCCTACC**GAGA**CTACTACCACTCCTACC | ETSTPTETTTTPT |
| r20 | **GAGA**CCTCTACTCCTACC**GAGA**CCACTACCACTCCTACC |  |
| r21 | **GAGA**CTACTACTCCTACC**GAGA**CTACTACCACTCCTACC |  |
| r24 | **GAGA**CCTCTACTCCTACC**GAGA**CTACTACTACTCCTACC |  |
| sr | **GAGA**CCTCTACTCCTACC | ETSTPT |
