# Supplementary material for: A New Aspergillus fumigatus Typing Method Based on Hypervariable Tandem Repeats Located within Exons of Surface Protein Coding Genes (TRESP)
Source: PLoS One. 2016 Oct 4;11(10):e0163869. doi: 10.1371/journal.pone.0163869 (PMC5049851; doi:10.1371/journal.pone.0163869)
Supplement: S4 Table — (DOCX) [file pone.0163869.s005.docx]

**S4 Table. CFEM repeat types: nucleotide and amino acid sequences identified among 175 *A. fumigatus* isolates.**

| CFEM Repeat type | Repeat sequence | Amino acid sequence |
| --- | --- | --- |
| r01 | TCCGGC | SG |
| r03 | TCTGGC |  |
| r04 | TCTGGT |  |
| r05 | TCGGGC |  |
| r07 | TCCGGT |  |
| r02 | TCCGAC | SD |
| r06 | TCTAGC | SS |
| r10 | TCTTCC |  |
| r13 | TCCAGC |  |
| r08 | TCCAAC | SN |
| r09 | TCTGCC | SA |
| r11 | ACCGCTACTGGC | TATG |
| r12 | GGTGCT | GA |
