# Supplementary material for: A New Aspergillus fumigatus Typing Method Based on Hypervariable Tandem Repeats Located within Exons of Surface Protein Coding Genes (TRESP)
Source: PLoS One. 2016 Oct 4;11(10):e0163869. doi: 10.1371/journal.pone.0163869 (PMC5049851; doi:10.1371/journal.pone.0163869)
Supplement: S5 Table — TRESP ID, number of strains of each type and proportion. (DOCX) [file pone.0163869.s006.docx]

**S5 Table. Total TRESP genotypes.** TRESP ID, number of strains of each type and proportion.

| TRESP ID | STRAIN NAME | TRESP GENOTYPE | TOTAL Nº OF STRAINS | AZOLE SUSCEPTIBILITY | PROPORTION (%) |
| --- | --- | --- | --- | --- | --- |
| 1 | CM5143 | t01m1.1c05A | 3 | S | 1.71 |
|  | CSP15 | t01m1.1c05A |  | S |  |
|  | TP24 | t01m1.1c05A |  | S |  |
| 2 | CM5888 | t01m1.1c08A | 4 | S | 2.29 |
|  | F45297 | t01m1.1c08A |  | R |  |
|  | CM7562 | t01m1.1c08A |  | S |  |
|  | T11 | t01m1.1c08A |  | R |  |
| 3 | TP22 | t01m1.1c09 | 1 | R | 0.57 |
| 4 | CM5360 | t01m1.1c12 | 1 | S | 0.57 |
| 5 | CM6003 | t01m1.1c17 | 1 | S | 0.57 |
| 6 | CM7510 | t01m1.1c18 | 1 | R | 0.57 |
| 7 | CM5163 | t01m1.7c08A | 1 | S | 0.57 |
| 8 | CM5393 | t01m3.1c08A | 1 | S | 0.57 |
| 9 | CM7468 | t01m3.2c05B | 1 | S | 0.57 |
| 10 | TP10 | t01m3.4c08A | 2 | R | 1.14 |
|  | TP32 | t01m3.4c08A |  | S |  |
| 11 | CM7396 | t01m3.4c16 | 1 | S | 0.57 |
| 12 | CM7401 | t01m3.4c19 | 1 | S | 0.57 |
| 13 | CM5485 | t01m3.4c20 | 1 | S | 0.57 |
| 14 | CM7477 | t01m3.5c05B | 1 | S | 0.57 |
| 15 | TP18 | t01m3.5c08A | 1 | S | 0.57 |
| 16 | CM4593 | t01m5.1c09 | 4 | R | 2.29 |
|  | CM5635 | t01m5.1c09 |  | S |  |
|  | CM7376 | t01m5.1c09 |  | S |  |
|  | TP23 | t01m5.1c09 |  | S |  |
| 17 | CSP19 | t01m5.2c08A | 1 | S | 0.57 |
| 18 | CM7555 | t01m5.3c05A | 2 | R | 1.14 |
|  | CSP1 | t01m5.3c05A |  | S |  |
| 19 | CM3248 | t01m5.3c06 | 1 | S | 0.57 |
| 20 | CM2161 | t01m5.3c07 | 2 | R | 1.14 |
|  | CSP12 | t01m5.3c07 |  | S |  |
| 21 | CM1244 | t01m5.3c08A | 1 | R | 0.57 |
| 22 | TP29 | t01m5.3c10 | 1 | S | 0.57 |
| 23 | CM7403 | t01m5.3c12 | 1 | S | 0.57 |
| 24 | TP14 | t01m5.3c17 | 1 | S | 0.57 |
| 25 | TP17 | t01m5.4c10 | 1 | S | 0.57 |
| 26 | CM2580 | t01m5.5c03 | 1 | S | 0.57 |
| 27 | CBS133.61 | t01m5.6c08A | 1 | S | 0.57 |
| 28 | CM5052 | t01m5.7c08A | 1 | S | 0.57 |
| 29 | CM2627 | t02m1.1c08A | 2 | R | 1.14 |
|  | CM5836 | t02m1.1c08A |  | S |  |
| 30 | CM2097 | t02m1.1c09 | 21 | R | 12.00 |
|  | CM2159 | t02m1.1c09 |  | R |  |
|  | CM2160 | t02m1.1c09 |  | R |  |
|  | CM3277 | t02m1.1c09 |  | R |  |
|  | CM3819 | t02m1.1c09 |  | R |  |
|  | CM4862 | t02m1.1c09 |  | S |  |
|  | CM4896 | t02m1.1c09 |  | S |  |
|  | CM6051 | t02m1.1c09 |  | S |  |
|  | CM7417 | t02m1.1c09 |  | S |  |
|  | CM7582 | t02m1.1c09 |  | R |  |
|  | M10731 | t02m1.1c09 |  | R |  |
|  | CM3279 | t02m1.1c09 |  | R |  |
|  | T22 | t02m1.1c09 |  | R |  |
|  | TP3 | t02m1.1c09 |  | S |  |
|  | TP4 | t02m1.1c09 |  | R |  |
|  | TP5 | t02m1.1c09 |  | R |  |
|  | TP6 | t02m1.1c09 |  | R |  |
|  | TP7 | t02m1.1c09 |  | R |  |
|  | TP8 | t02m1.1c09 |  | R |  |
|  | TP9 | t02m1.1c09 |  | R |  |
|  | CM4211 | t02m1.1c09 |  | R |  |
| 31 | CM2164 | t02m1.2c04 | 1 | R | 0.57 |
| 32 | CM5536 | t02m1.2c09 | 2 | S | 1.14 |
|  | CM5703 | t02m1.2c09 |  | S |  |
| 33 | CM7609 | t02m1.4c09 | 1 | R | 0.57 |
| 34 | CM3272 | t02m1.5c09 | 2 | R | 1.14 |
|  | CM3271 | t02m1.5c09 |  | R |  |
| 35 | CM5054 | t02m1.8c09 | 2 | S | 1.14 |
|  | CM7496 | t02m1.8c09 |  | S |  |
| 36 | CM5390 | t02m5.4c09 | 1 | S | 0.57 |
| 37 | TP15 | t03m1.1c04 | 1 | S | 0.57 |
| 38 | CM5417 | t03m1.1c05A | 1 | S | 0.57 |
| 39 | CM2202 | t03m1.1c07 | 1 | S | 0.57 |
| 40 | AF237 | t03m1.1c08A | 3 | S | 1.71 |
|  | CM5917 | t03m1.1c08A |  | S |  |
|  | CM6052 | t03m1.1c08A |  | S |  |
| 41 | CM2266 | t03m1.1c08B | 3 | R | 1.71 |
|  | CM3500 | t03m1.1c08B |  | R |  |
|  | CM6335 | t03m1.1c08B |  | S |  |
| 42 | CM2120 | t03m1.1c09 | 1 | S | 0.57 |
| 43 | CM5403 | t03m1.1c12 | 1 | S | 0.57 |
| 44 | CM6458 | t03m1.3c04 | 1 | S | 0.57 |
| 45 | TP2 | t03m1.3c05A | 1 | S | 0.57 |
| 46 | CM5929 | t03m1.6c07 | 1 | S | 0.57 |
| 47 | CM2144 | t03m5.3c05A | 1 | R | 0.57 |
| 48 | TP21 | t03m5.3c08A | 1 | S | 0.57 |
| 49 | CM5325 | t03m5.3c09 | 1 | S | 0.57 |
| 50 | CM5178 | t03m5.4c08A | 1 | S | 0.57 |
| 51 | CM2495 | t03m9.1c13 | 1 | S | 0.57 |
| 52 | CM5297 | t04Am1.1c02 | 1 | S | 0.57 |
| 53 | CM5409 | t04Am1.1c03 | 1 | S | 0.57 |
| 54 | CM6031 | t04Am1.1c05A | 3 | S | 1.71 |
|  | CM6450 | t04Am1.1c05A |  | S |  |
|  | CM7470 | t04Am1.1c05A |  | S |  |
| 55 | CM2162 | t04Am1.1c08A | 5 | R | 2.86 |
|  | CM2163 | t04Am1.1c08A |  | R |  |
|  | CM4876 | t04Am1.1c08A |  | S |  |
|  | CM5590 | t04Am1.1c08A |  | S |  |
|  | TP13 | t04Am1.1c08A |  | S |  |
| 56 | TP20 | t04Am1.1c08B | 2 | R | 1.14 |
|  | TP25 | t04Am1.1c08B |  | S |  |
| 57 | CM4599 | t04Am1.1c09 | 1 | R | 0.57 |
| 58 | CM6126 | t04Am1.1c10 | 1 | S | 0.57 |
| 59 | CM5410 | t04Am1.1c12 | 1 | S | 0.57 |
| 60 | CM5973 | t04Am1.1c19 | 1 | S | 0.57 |
| 61 | CM5756 | t04Am1.3c08A | 2 | S | 1.14 |
|  | CM6108 | t04Am1.3c08A |  | S |  |
| 62 | CM7632 | t04Am1.9c01 | 1 | S | 0.57 |
| 63 | CM7473 | t04Am1.9c08A | 1 | S | 0.57 |
| 64 | CM5489 | t04Am3.4c08A | 2 | S | 1.14 |
|  | CM7393 | t04Am3.4c08A |  | S |  |
| 65 | CM5411 | t04Am3.4c09 | 1 | S | 0.57 |
| 66 | TP26 | t04Am3.4c16 | 1 | S | 0.57 |
| 67 | CM6073 | t04Am3.6c08A | 1 | S | 0.57 |
| 68 | CM7408 | t04Am3.7c08A | 1 | S | 0.57 |
| 69 | CM7397 | t04Am4.1c08A | 1 | S | 0.57 |
| 70 | TP19 | t04Am4.2c16 | 1 | S | 0.57 |
| 71 | CM796 | t04Am5.3c06 | 1 | R | 0.57 |
| 72 | CM1245 | t04Am5.3c08A | 3 | R | 1.71 |
|  | CM1252 | t04Am5.3c08A |  | R |  |
|  | CM2158 | t04Am5.3c08A |  | R |  |
| 73 | CM5757 | t04Am5.3c16 | 1 | S | 0.57 |
| 74 | CM7405 | t04Am5.4c08A | 3 | S | 1.71 |
|  | TP27 | t04Am5.4c08A |  | S |  |
|  | TP28 | t04Am5.4c08A |  | S |  |
| 75 | CM7410 | t04Am5.5c03 | 1 | S | 0.57 |
| 76 | CM5907 | t04Am5.5c08A | 3 | S | 1,71 |
|  | CM6119 | t04Am5.5c08A |  | S |  |
|  | CM7250 | t04Am5.5c08A |  | S |  |
| 77 | CM4594 | t04Bm1.1c04 | 2 | R | 1.14 |
|  | CM3820 | t04Bm1.1c04 |  | R |  |
| 78 | R13 | t04Bm1.2c04 | 1 | R | 0.57 |
| 79 | CM4050 | t04Bm1.2c12 | 1 | R | 0.57 |
| 80 | CSP4 | t05m1.1c05A | 2 | S | 1.14 |
|  | CSP8 | t05m1.1c05A |  | S |  |
| 81 | CM7399 | t05m5.3c10 | 2 | S | 1.14 |
|  | CM7400 | t05m5.3c10 |  | S |  |
| 82 | CM5916 | t05m5.4c05A | 1 | S | 0.57 |
| 83 | CSP6 | t06Am1.1c09 | 1 | S | 0.57 |
| 84 | AF293 | t06Am2.1c01 | 1 | S | 0.57 |
| 85 | CM5725 | t06Bm3.4c08A | 2 | S | 1.14 |
|  | CM5736 | t06Bm3.4c08A |  | S |  |
| 86 | CM5419 | t06Bm6.2c08A | 1 | S | 0.57 |
| 87 | CM7407 | t06Bm6.3c04 | 1 | S | 0.57 |
| 88 | CM2733 | t08m7.1c11 | 3 | S | 1.71 |
|  | CM4602 | t08m7.1c11 |  | S |  |
|  | CM7570 | t08m7.1c11 |  | S |  |
| 89 | CM3249 | t08m9.1c14 | 1 | S | 0.57 |
| 90 | CM3262 | t08m9.1c15 | 1 | S | 0,57 |
| 91 | CM6422 | t09m1.1c08A | 1 | S | 0,57 |
| 92 | CM6413 | t09m5.3c10 | 1 | S | 0,57 |
| 93 | TP1 | t10m1.1c05A | 1 | S | 0,57 |
| 94 | CM7467 | t11m1.1c08A | 2 | S | 1.14 |
|  | TP16 | t11m1.1c08A |  | S |  |
| 95 | CM3936 | t11m1.2c04 | 2 | R | 1.14 |
|  | T18 | t11m1.2c04 |  | R |  |
| 96 | CM4023 | t11m1.2c09 | 2 | R | 1.14 |
|  | CM6616 | t11m1.2c09 |  | S |  |
| 97 | CM3273 | t11m1.5c09 | 1 | R | 0.57 |
| 98 | TP31 | t13m3.4c03 | 1 | S | 0.57 |
| 99 | CM4592 | t13m7.1c11 | 1 | S | 0.57 |
| 100 | TP11 | t14m2.2c02 | 2 | S | 1.14 |
|  | TP12 | t14m2.2c02 |  | S |  |
| 101 | CM7398 | t14m3.4c01 | 1 | S | 0.57 |
| 102 | CM7402 | t14m3.4c19 | 1 | S | 0.57 |
| 103 | CM2730 | t15m7.1c11 | 1 | S | 0.57 |
| 104 | CM5621 | t18Am1.1c05A | 1 | S | 0.57 |
| 105 | TP30 | t18Am5.3c10 | 1 | S | 0.57 |
| 106 | CM6448 | t18Bm6.1c08A | 1 | S | 0.57 |
| 107 | CM4946 | t19m8.1c11 | 1 | S | 0.57 |
| 108 | CM7560 | t19m9.1c13 | 1 | S | 0.57 |
| 109 | CM5392 | t25m1.1c08A | 1 | S | 0.57 |
| 110 | CM4982 | t26m1.1c08A | 1 | S | 0.57 |
| 111 | CM7009 | t27m10.1c21 | 1 | S | 0.57 |
| Total |  | | 175 |  | 100,00 |

S, susceptible; R, resistant
