# Supplementary material for: A New Aspergillus fumigatus Typing Method Based on Hypervariable Tandem Repeats Located within Exons of Surface Protein Coding Genes (TRESP)
Source: PLoS One. 2016 Oct 4;11(10):e0163869. doi: 10.1371/journal.pone.0163869 (PMC5049851; doi:10.1371/journal.pone.0163869)
Supplement: S6 Table — (DOCX) [file pone.0163869.s007.docx]

**S6 Table.** **GenBank Accesion Numbers of all TRESP types.**

| Strain and target_ID | Genotype | GenBank Acc. Ns |
| --- | --- | --- |
| CM_CBS_CSP | t01 | KX787551 |
| CM_4862_CSP | t02 | KX787552 |
| CM_5917_CSP | t03 | KX787553 |
| CM_6450_CSP | t04A | KX787554 |
| CM_R13_CSP | t04B | KX787555 |
| CM_5916_CSP | t05 | KX787556 |
| CM_AF293_CSP | t06A | KX787557 |
| CM_5419_CSP | t06B | KX787558 |
| CM_2733_CSP | t08 | KX787559 |
| CM_6413_CSP | t09 | KX787560 |
| CM_15.2_CSP | t10 | KX787561 |
| CM_3273_CSP | t11 | KX787562 |
| CM_4592_CSP | t13 | KX787563 |
| CM_7398_CSP | t14 | KX787564 |
| CM_2730_CSP | t15 | KX787565 |
| CM_5621_CSP | t18A | KX787566 |
| CM_6448_CSP | t18B | KX787567 |
| CM_4946_CSP | t19 | KX787568 |
| CM_5392_CSP | t25 | KX787569 |
| CM_4982_CSP | t26 | KX787570 |
| CM_7009_CSP | t27 | KX787571 |
| CM_AF237_MP2 | m1.1 | KX787517 |
| CM_5536_MP2 | m1.2 | KX787518 |
| CM_5756_MP2 | m1.3 | KX787519 |
| CM_7609_MP2 | m1.4 | KX787520 |
| CM_3273_MP2 | m1.5 | KX787521 |
| CM_5929_MP2 | m1.6 | KX787522 |
| CM_5163_MP2 | m1.7 | KX787523 |
| CM_5054_MP2 | m1.8 | KX787524 |
| CM_7632_MP2 | m1.9 | KX787525 |
| CM_AF293_MP2 | m2.1 | KX787526 |
| CM_TP11_MP2 | m2.2 | KX787527 |
| CM_5393_MP2 | m3.1 | KX787528 |
| CM_7468_MP2 | m3.2 | KX787529 |
| CM_7560_MP2 | m3.3 | KX787530 |
| CM_7393_MP2 | m3.4 | KX787531 |
| CM_7477_MP2 | m3.5 | KX787532 |
| CM_6073_MP2 | m3.6 | KX787533 |
| CM_7408_MP2 | m3.7 | KX787534 |
| CM_7397_MP2 | m4.1 | KX787535 |
| CM_TP19_MP2 | m4.2 | KX787536 |
| CM_5635_MP2 | m5.1 | KX787537 |
| CM_CSP19_MP2 | m5.2 | KX787538 |
| CM_5325_MP2 | m5.3 | KX787539 |
| CM_5390_MP2 | m5.4 | KX787540 |
| CM_2580_MP2 | m5.5 | KX787541 |
| CM_CBS_MP2 | m5.6 | KX787542 |
| CM_5052_MP2 | m5.7 | KX787543 |
| CM_6448_MP2 | m6.1 | KX787544 |
| CM_5419_MP2 | m6.2 | KX787545 |
| CM_7407_MP2 | m6.3 | KX787546 |
| CM_2733_MP2 | m7.1 | KX787547 |
| CM_4946_MP2 | m8.1 | KX787548 |
| CM_2495_MP2 | m9.1 | KX787549 |
| CM_7009_MP2 | m10.1 | KX787550 |
| CM_AF293_CFEM | c01 | KX787494 |
| CM_TP12_CFEM | c02 | KX787495 |
| CM_7410_CFEM | c03 | KX787496 |
| CM_TP15_CFEM | c04 | KX787497 |
| CM_5143_CFEM | c05A | KX787498 |
| CM_7468_CFEM | c05B | KX787499 |
| CM_3248_CFEM | c06 | KX787500 |
| CM_2202_CFEM | c07 | KX787501 |
| CM_AF237_CFEM | c08A | KX787502 |
| CM_TP25_CFEM | c08B | KX787503 |
| CM_2120_CFEM | c09 | KX787504 |
| CM_7399_CFEM | c10 | KX787505 |
| CM_2730_CFEM | c11 | KX787506 |
| CM_4050_CFEM | c12 | KX787507 |
| CM_7560_CFEM | c13 | KX787508 |
| CM_3249_CFEM | c14 | KX787509 |
| CM_3262_CFEM | c15 | KX787510 |
| CM_7396_CFEM | c16 | KX787511 |
| CM_6003_CFEM | c17 | KX787512 |
| CM_7510_CFEM | c18 | KX787513 |
| CM_7401_CFEM | c19 | KX787514 |
| CM_5485_CFEM | c20 | KX787515 |
| CM_7009_CFEM | c21 | KX787516 |
